# Relationships Between the Microvascular Network and Mast Cell Density in Malignant Melanoma

**DOI:** 10.3390/medicina62040752

**Published:** 2026-04-14

**Authors:** Victor Cristian Dumitrascu, Amalia Raluca Ceausu, Cristina Raluca Mihulecea, Florica Sandru, Adina Octavia Duse, Alexandra Laura Mederle, Maria Rotaru, Tiberiu Bratu, Marius Raica, Roxana Popescu, Nela Pusa Gaje

**Affiliations:** 1 Doctoral Studies, “Victor Babeș” University of Medicine and Pharmacy, 300041 Timișoara, Romania; 2 Department of Surgery, Discipline of Plastic Surgery, “Victor Babeș” University of Medicine and Pharmacy, 300041 Timișoara, Romania; 3 Department of Microscopic Morphology, Histology, Angiogenesis Research Center, “Victor Babeș” University of Medicine and Pharmacy, 300041 Timișoara, Romania; 4 Dermatovenerology Clinic, Emergency County Clinical Hospital, 550245 Sibiu, Romania; 5 Faculty of Medicine, “Lucian Blaga” University, 550169 Sibiu, Romania; 6 Department of Dermatology, University of Medicine and Pharmacy “Carol Davila”, 020021 Bucharest, Romania; 7 Elias University Emergency Hospital, 011461 Bucharest, Romania; 8 Department of Balneology, Medical Rehabilitation and Rheumatology, Clinic of Physical Medicine, Balneology and Rheumatology, Transdisciplinary Research Center in Medical Rehabilitation, Balneology and Rheumatology Research Center, 300041 Timisoara, Romania; 9 Department of Dermatology, “Victor Babeș” University of Medicine and Pharmacy, 300041 Timișoara, Romania; 10 Department of Microscopic Morphology, Discipline of Cellular and Molecular Biology, “Victor Babeș” University of Medicine and Pharmacy, 300041 Timișoara, Romania

**Keywords:** melanoma, mast cell, angiogenesis, microvascular density

## Abstract

*Background and Objectives*: Angiogenesis plays an important role in many types of cancers, including melanoma. Mast cells are among the most important cellular partners involved in the angiogenic process. The purpose of the present study is to establish the interrelations between microvessels and mast cell density, and to describe the prognostic role of mast cells in malignant melanoma. *Materials and Methods*: A total of 92 cases of melanoma were evaluated. Hematoxylin–eosin staining and CD34/mast cell tryptase double immunostaining were performed. *Results*: Of the cases, 28 were classified as T1 (30.43%), 31 as T2 (33.69%), 21 as T3 (22.82%), and 12 as T4 (13.04%). Mast cells were mainly located in the infiltrate area in cases with inflammatory infiltrate. Few mast cells were observed in the absence of infiltrate. A granular type was observed in intratumoral areas with an isolated and perivascular distribution of mast cells. A significant correlation was found between intratumoral MVD and intratumoral MCD (mast cell density) in T1 and T4 melanoma cases (*p* = 0.0001 and *p* = 0.0029, respectively). In T2 cases, significant correlations were found between intratumoral and peritumoral MVD and G (p = 0.0169, *p* = 0.0003), between intratumoral MCD and MVD (*p* = 0.0041), and between peritumoral MVD and the number of peritumoral mast cells (*p* = 0.0011). T3 cases showed a significant correlation between the density of intratumoral mast cells and peritumoral ones (*p* = 0.001); between the MVD and mast cell density of the intratumoral area (*p* = 0.0001); between the intratumoral MVD and peritumoral mast cell density (*p* = 0.0001); between the MVD and mast cell density of the peritumoral area (*p* = 0.0325); and between the peritumoral MVD and intratumoral mast cell density (*p* = 0.0458). *Conclusions*: Both intratumoral and peritumoral mast cells were found to participate in melanoma progression, and they showed greater involvement in the early phases of angiogenesis in malignant melanoma.

## 1. Introduction

Malignant melanoma is the 19th most common cancer diagnosis worldwide, and one of the most aggressive [1]. At the skin level, melanoma is the third most common malignancy after basal and squamous cell carcinoma. Despite representing less than 5% of skin cancers, it is responsible for 65% of skin cancer-related deaths [2,3].

The standard procedure for microscopic diagnosis is histologic examination. From a histological point of view, melanomas are characterized by greater heterogeneity and numerous morphological variants [4]. The microscopic prognostic elements are limited and debatable.

Different cells are involved in the interrelations between melanoma and the tumor microenvironment, including mast cells [5]. Mast cells can play a dual role in the stages of melanoma appearance, growth, and metastasis by releasing pro-inflammatory and immune-inhibitory mediators [6]. Their involvement in angiogenesis and tumor progression in melanoma has been demonstrated in various experimental models, including murine ones [7].

Described by Warren and Shubik in 1966 [8], tumor angiogenesis is essential in melanoma, especially during the vertical growth phase. From an angiogenic point of view, only a few mechanisms have been described, including sprouting, intussusception, and vasculogenic mimicry. Vasculogenesis has also been observed [9,10].

Due to their morphological and functional heterogeneity, mast cells are important actors in the process of angiogenesis, releasing different angiogenic mediators [11] and supporting endothelial cell proliferation [12].

The duality of mast cell density has been reported across different tumor types, including in melanoma. Tumor types associated with increased mast cell density values include Hodgkin’s lymphoma; giant cell tumor of bone [13]; and those of digestive origin, such as colorectal, pancreatic, and esophageal cancer [14,15,16]. Duality is observed in the case of melanomas, where some studies have reported a severe prognosis accompanied by increased MC density [17,18], while others have reported decreased MC density [19,20].

Mast cells have been observed in the microenvironment of many tumor types. Although this phenomenon has been well known for many years, the real role of MCs in the progression and spread of the tumor is less well understood. Mast cells have been shown to be involved in oral squamous carcinoma, esophageal cancer, gastric cancer, pancreatic ductal adenocarcinoma, breast cancer, bladder cancer, liver cancer, cervical carcinoma, melanoma, thyroid cancer, colorectal and prostatic cancer, and renal cell carcinoma. In other cancers, such as NSCLC, the role of mast cells is controversial. Based on these premises, the aim of the present study was to evaluate the interrelation between the microvascular network and mast cell density in malignant melanoma.

## 2. Materials and Methods

The present study included 92 cases from three different university centers (’Carol Davila’ University of Medicine and Pharmacy, Bucharest, ’Lucian Blaga’ University, Faculty of Medicine, Sibiu and ’Victor Babeș’ University of Medicine and Pharmacy, Timișoara). They were compared not only from a molecular point of view, but also in terms of the distribution and particularities of the geographic area. The present study was conducted in accordance with The Declaration of Helsinki and was approved by the Ethics Committees of the Victor Babeș University of Medicine and Pharmacy (approval no. 28/23 September 2019 from 10 March 2022; approval no.46/20 June 2022 Timișoara, Romania), The Emergency Hospital of Timis County (approval no. 347/1 November 2022), the Emergency Hospital of Sibiu County (approval no. 6001/9 March 2022; Sibiu, Romania), and the Local Ethics Committee of the Bucharest University Emergency Hospital no. 37003/17 June 2022. Adult patients diagnosed with melanoma were included in the study. Patients were excluded if they were minors, or if they had non-melanocytic tumors or common nevi.

Morphological evaluation was performed on sections stained with the hematoxylin–eosin method, using a standard technique with the Leica automatic system. The histopathological type of melanoma, T parameter, pigmentation, emboli, mitosis, and the degree of differentiation were established by examination of these sections.

Double CD34/mast cell tryptase immunostaining was used. This method is useful for identifying vascular and mast cell density. The first part of the immunohistochemical technique consists of heat-induced epitope retrieval. For this, we used ready-to-use Bond Epitope Retrieval Solution 2 from Leica Biosystems (Newcastle upon Tyne, UK). This stage was followed by endogenous peroxidase blocking. Five minutes after application, slides were incubated with primary antibody, then incubated for 20 min with CD34. Mouse anti-human monoclonal antibody was used, and the clone was QBEnd10. Visualization was conducted using The Bond Polymer Refine Detection System components: mainly the post-primary antibody for 8 min and polymer for 8 min. Chromogen was then applied (3.3-diaminobenzidine dihydrochloride for 10 min), followed by application of the peroxide block (5 min), incubation with second antibody mast cell tryptase ((MCT) Leica Bond, RTU, mouse anti-human monoclonal antibody, clone 10D11) for 20 min, application of post primary alkaline phosphatase (AP) for 20 min, Polymer AP for 20 min, Mixed Red Refine for 10 min, Mixed Red Refine for 5 min, and Hematoxylin for 5 min. This was carried out with the Bond Max Autostainer (Leica Biosystem). Then, the slides were mounted and microscopically assessed.

The morphologically and immunohistochemically stained sections were analyzed using Zeiss Axiocam 506 microscopes (Jena, Germany) and a Nikon AY260. The Weidner standard method was used to evaluate the microvascular density [21]. Three microscopic fields with maximum vascular density and mast cell density were assessed at magnification ×200. Mast cells were stained in red and blood vessels in brown. Results were taken as the arithmetic mean. For each slide, the tumor and peritumor area were evaluated. Statistical analysis was performed using the MedCalc software (version 12.6, MedCalc Software). The Pearson method was used. A *p*-value of <0.05 was considered statistically significant, while a *p*-value of <0.001 was considered strongly statistically significant. Charts and correlation tables were automatically generated by the MedCalc software.

## 3. Results

The assessment of the cases after morphological staining revealed 92 cases which were diagnosed as melanoma. The following parameters were used for evaluation: T, G, presence of inflammatory infiltrate, presence of mitosis, emboli, presence of pigment, microvascular density in the intratumoral and peritumoral areas, and mast cell density in the intratumoral and peritumoral zones. In order to maintain blinding, the outcome assessor was not involved in delivering the intervention, and was blinded to the tumor and adjacent tissue status.

**Figure 1:**
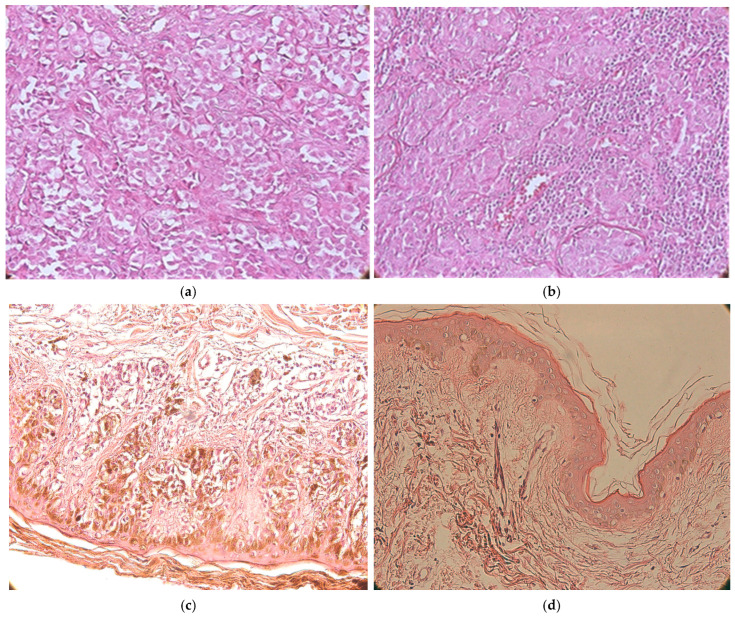
(**a**) Malignant melanoma. Tumor cells with round and oval shapes, large pleomorphic nuclei, prominent nucleoli, eosinophilic cytoplasm; magnification ×400, hematoxylin and eosin stain. (**b**) Presence of inflammatory infiltrate around nests of malignant cells; magnification ×400, hematoxylin and eosin staining. (**c**) Malignant melanoma, T1, G1, numerous melanophages, low inflammatory infiltrate, magnification ×400, hematoxylin and eosin staining. (**d**) Lentigo maligna melanoma (T1, G1), without inflammatory infiltrate, emboli, mitoses, or pigment; magnification ×200, hematoxylin and eosin staining.

Microscopically, the malignant melanomas were composed of round, oval, or spindle-shaped cells, with large, pleomorphic nuclei (Figure 1a). Typical and atypical mitoses were present in most cases. The melanin content was variable, and in some cases it was absent. In some cases, the tumor cells exhibited a spindle-shaped appearance, forming sarcomatoid patterns.

In some cases, the presence of an inflammatory infiltrate was observed around the nests of tumor cells, consisting mainly of lymphocytes and plasma cells, as shown in Figure 1b. The presence of pigment was used to evaluate the images; in some cases, we observed the presence of a large number of melanophages, which appear brown when assessed morphologically (Figure 1c).

Lentigo malignant melanoma was characterized by an increased number of melanocytes arranged along the dermoepidermal junction. The malignant melanocytes were arranged in small nests (lentiginous pattern) or nests arranged horizontally or confluently, which showed variable sizes and shapes (nevus/dysplastic pattern) (Figure 1d).

**Figure 2:**
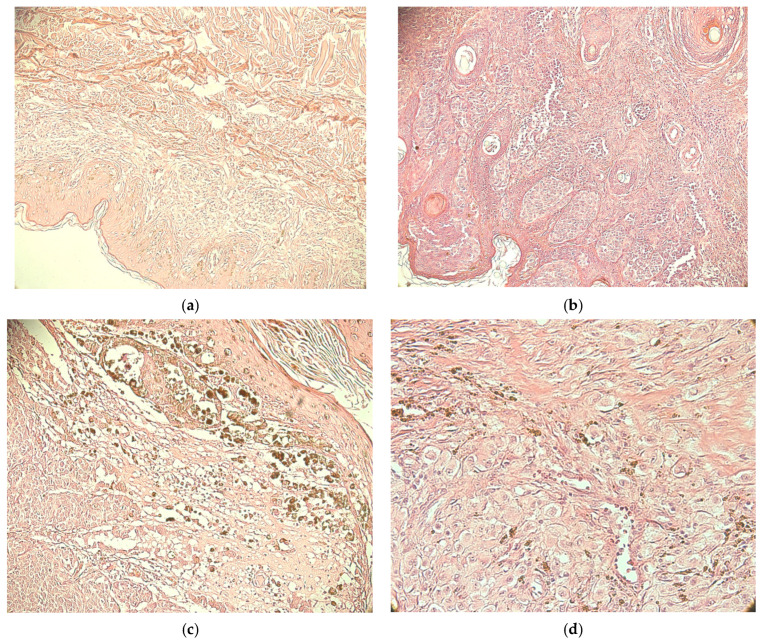
Histopathological aspects of malignant melanomas. (**a**) T1, (**b**) T2, (**c**) T3, and (**d**) T4, hematoxylin and eosin staining.

From the 92 cases of melanoma included in this study, 28 cases were classified as T1 (30.43%), 31 as T2 (33.69%), 21 as T3 (22.82%), and 12 as T4 (13.04%) (Figure 2).

The T1-classified cases were assessed for emboli, the presence of melanin pigment, and inflammatory infiltrate. Inflammatory infiltrate was observed in six cases. The value score was +1 (five cases) and +2 (one case). Emboli were absent in all cases. Mitoses were observed in only one case; pigment was present in seven cases; and all 28 cases were graded as G1.

Emboli were absent in all cases classified as T2. Mitoses were observed in only nine cases. Melanin pigment was present in 11 cases (seven cases scored as value 1, and three cases quantified with score 2).

The group of T3-classified cases was characterized by the absence of emboli in G1 and G2 cases and presence in a single G3 case. Mitoses were present in 1 G1 case, 15 G2 cases, and 2 G3 cases. Pigment was absent in G1 cases, and present in ten G2-classified cases and three G3 cases. The distribution of inflammatory infiltrate according to the cases in this group is detailed in Table 1.

Our observations are summarized in Table 1, below.

The distribution and clinical–pathological variables of cases from the Bucharest University Center are summarized in Table 2.

The distribution and clinical parameters of cases from the Sibiu and Timișoara Centers are summarized in Table 3 and Table 4.

The overall survival rate at five years for evaluated patients was 93.47%.

**Figure 3:**
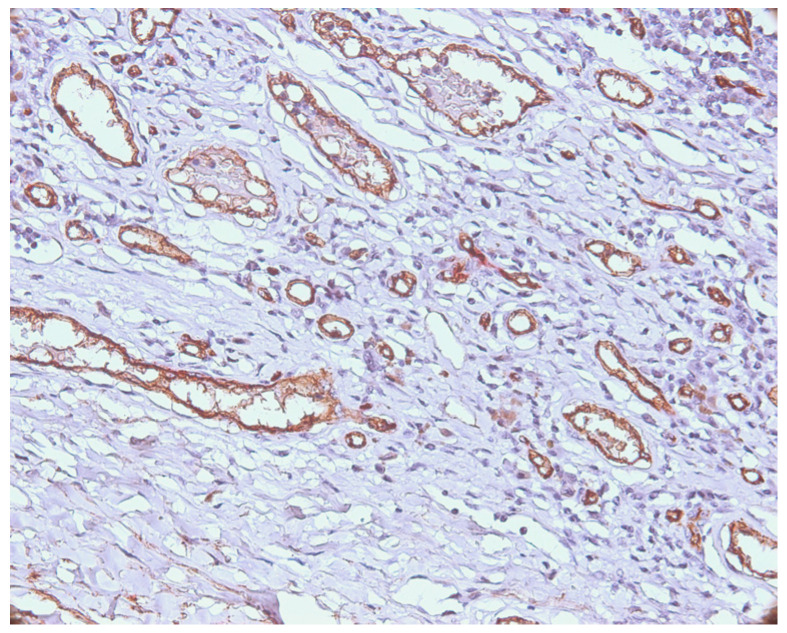
CD34/MCT double immunostaining: presence of intravascular tumor cells in one case of melanoma, ×200 magnification.

The presence of emboli was noticed on HE staining, as well as on the double immunostaining CD34/MCT (Figure 3).

The CD34/MCT double immunostaining allowed for the evaluation of the number and distribution patterns of MCs in intra- and peritumoral areas. Mast cells were absent from the intratumoral area in 27 cases (26.21% of cases) and present in the rest (73.78% of cases). A particularly low number of MCs, and sometimes complete absence, was observed in lymph node metastases.

**Figure 4:**
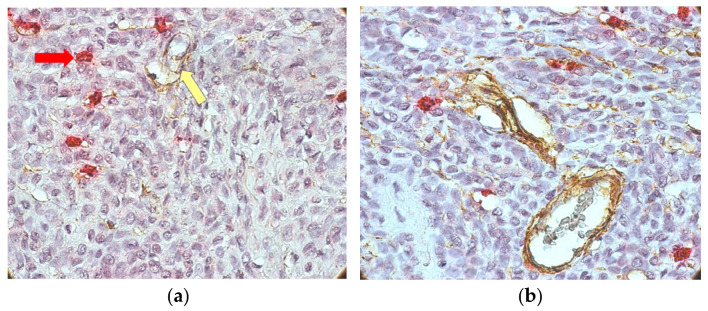
CD34/MCT double immunostaining (mast cells: red arrow; blood vessels: yellow arrow)—an isolated and perivascular distribution pattern of mast cells in intratumoral areas at ×400 magnification (**a**) and ×200 magnification (**b**).

In cases with inflammatory infiltrate and many melanophages, MCs were located in the infiltrate area. In the absence of infiltrate, few MCs were observed. More MCs were detected in the areas surrounding the melanoma, as well as a possible tendency for the intraepithelial migration of MCs. An isolated and perivascular distribution pattern of MCs was observed in intratumoral areas (Figure 4a,b). Among the main histological types of MCs, granular types predominated.

The double immunoreaction CD34/MCT revealed the presence of a numerous MCs and blood vessels in early stages of melanoma development compared to stages T3 and T4. The average values of vessels and MCs associated with each tumor stage are detailed below.

**Figure 5:**
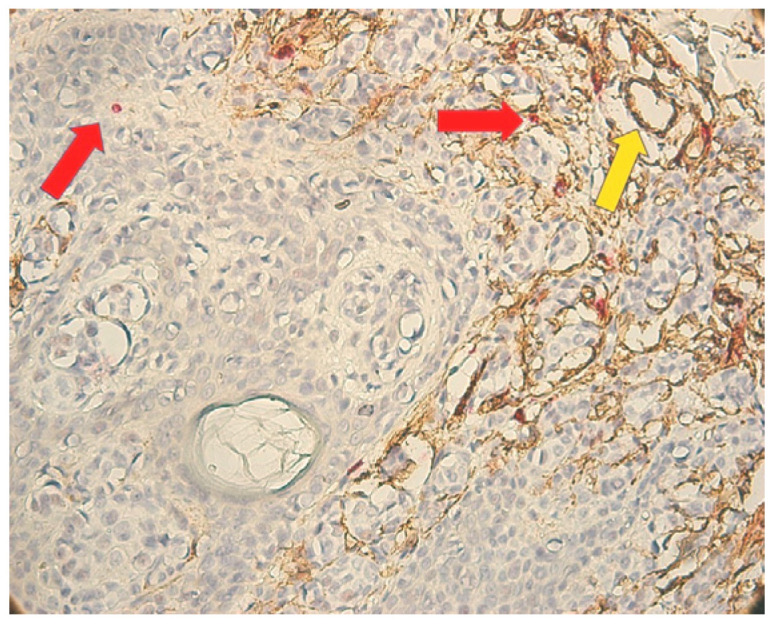
CD34/MCT double immunostaining. The distribution and density of mast cells (red arrows) and blood vessels (yellow arrow) in T1 melanoma case: ×200 magnification.

For T1 cases, the highest mast cell value in the intratumoral area was 24, and in the peritumoral area, 49. The highest values of microvascular density (MVD) were 42 in intratumoral areas and 23 in peritumoral areas. The distribution and density of mast cells and blood vessels is shown in Figure 5.

IQR values for the intratumoral mast cell density and T1 cases were 1.33 and 5.67 in the peritumoral area. IQR values for intratumoral and peritumoral MVD were 1.5 and 5.67. The mean and standard deviation values of MCD were 2.304 ± 5.52 (intratumoral) and 13.34 ± 12.76 (peritumoral). The MVD values were 3.87 ± 9.45 (intratumoral) and 10.34 ± 5.75 (peritumoral).

**Figure 6:**
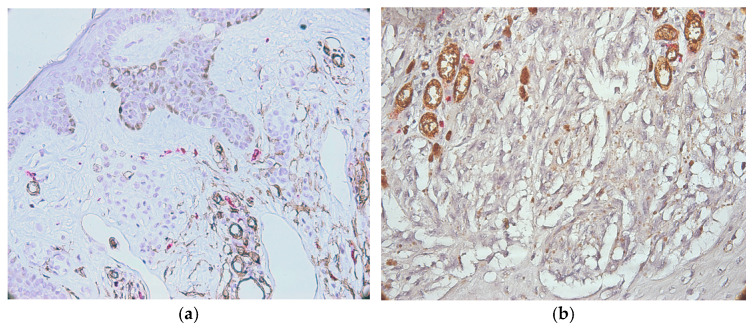
CD34/MCT double immunostaining in well ((**a**), ×100 magnification) and moderately ((**b**), ×200 magnification) differentiated T2 melanoma.

Cases evaluated as T2 and well differentiated presented the following highest values for MCD and MVD: 8 and 2.33 for the intratumoral area and 23.33 and 9.66 for the peritumoral area. The mean and standard deviation values were 2.89± 2.58 and 1.03 ± 0.65 (intratumoral MCD and MVD, respectively). Values of 13.46 ± 4.93 and 8.13 ± 1.72 (peritumoral MCD and MVD) were noticed. The IQR values varied between 0.66 and 2.33 (for MVD and MCD intratumoral) and 2.66 and 5 (for MCD and MVD peritumoral). In T2 but moderately differentiated cases, we observed results of 14 for MCs and 24.33 for MVD from the intratumoral area. The highest values found in the peritumoral area were 27 for MCs and 24.33 for MVD. The IQR values were 7 for MCD and 17.67 for MVD (intratumoral area) and 12.34 and 3 (MCD and MVD values in peritumoral zones). Values of 4.80 ± 5.97 and 15.63 ± 7.15 were found for MCD in intra- and peritumoral areas, respectively. The microvascular density was 7.52 ± 9.05 in the intratumoral area and 15.33 ± 4.18 in the peritumoral area. This is shown in Figure 6.

**Figure 7:**
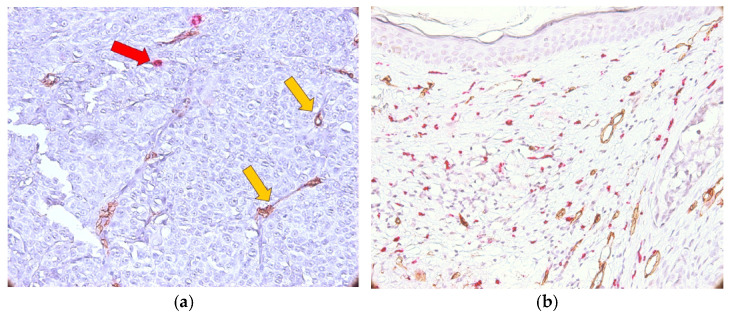
CD34/MCT double immunostaining in T3 melanoma cases: MVD (yellow arrows) and MCD (red arrow) in intratumoral ((**a**)—×200 magnification) and peritumoral areas ((**b**)—×200 magnification).

Cases evaluated as T3 had the following highest values of mast cells: 16.33 (intratumoral area) and 17.66 (peritumoral area). The highest values of MVD in the intratumoral area (20) were close to those from the peritumoral area (21.66). The IQR for MCD was 6.66 in the peritumoral area, and for intratumoral MVD it was 5.16. The IQR for MCD was 1.66 inside of the tumoral area, and for MVD in the peritumoral zone it was 2.66. The following values were observed: 3.68 ± 7.49 (MCD intratumoral); 12.64 ± 4.63 (MCD peritumoral); 5.64 ± 4.40 (MVD intratumoral); and 15.79 ± 3.07 (MVD peritumoral). The distribution of mast cells and blood vessels in the intratumoral area (a) and peritumoral area (b) are shown in Figure 7 [22]. The main mechanisms of tumor angiogenesis were intussusception and sprouting. Blood vessels with and without a lumen were observed.

**Figure 8:**
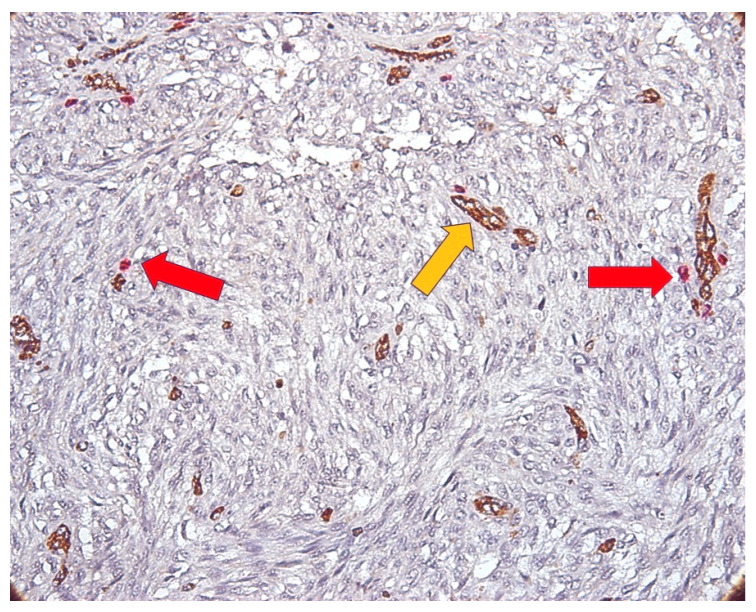
CD34/MCT double immunostaining in T4 melanoma cases, MVD and MCD in intratumoral area (×400 magnification), red arrows (mast cells) and yellow arrows (blood vessels).

In melanoma cases evaluated as T4, the highest value of MC density in the intratumoral area was 6.33, and in the peritumoral area, it was 12.33. The highest values of MVD for assessed cases were 13.66 (intratumoral) and 17.66 (peritumoral). In terms of IQR, the values for MCD were 2.34 (intratumoral) and 5.67 (peritumoral) and the values for MVD were 8.33 (intratumoral) and 7.34 (peritumoral). The mean and standard deviation for MCD were 2.027 ± 1.89 (intratumoral) and 9.93 ± 4.97 (peritumoral). The values for MVD were 7.02 ± 4.37 (intratumoral) and 11.99 ± 3.52 (peritumoral). The type, morphology, and distribution of the mast cells and blood vessels are shown in Figure 8.

For T1 melanoma cases, a significant correlation was found between peritumoral MVD and peritumoral mast cell density (*p* = 0.0001) and between intratumoral MVD and intratumoral MCD (*p* = 0.0001). The last correlation was found for T4 cases with a value of *p* = 0.0029. For T2 cases, a significant correlation was found between intratumoral MVD and G (*p* = 0.0169), and between intratumoral MCD and MVD (*p* = 0.0041). For these cases, a significant correlation was found between peritumoral MVD and the number of peritumoral mast cells (*p* = 0.0011), and between peritumoral MVD and G (*p* = 0.0003).

The T3 cases showed a significant correlation between the density of intratumoral mast cells and peritumoral ones (*p* = 0.001); between intratumoral MVD and intratumoral MCD (*p* = 0.0001); between intratumoral MVD and peritumoral MCD (*p* = 0.0001); between peritumoral MVD and peritumoral MCD (*p* = 0.0325); and between peritumoral MVD and intratumoral mast cell density (*p* = 0.0458).

## 4. Discussion

In this study, we have demonstrated that angiogenesis remains an important factor in the progression of melanoma cases from three different university centers. The main cellular partners are cancer-associated fibroblasts, mast cells, and melanoma-associated macrophages [23]. We observed the presence of mast cells with distinctive distribution characteristics in the intra- and peritumoral areas of melanomas.

By using the immunohistochemistry method, Rajabi et al. [24] evaluated the distribution of MCs and reported the values of mean and standard deviation for mast cells in stage 1 as 9.4 ± 4.2 (intratumoral) and 13.4 ± 2.4 (peritumoral). In our study, the following values of MCD were found: 2.30± 5.52 (intratumoral) and 13.34 ± 12.7 (peritumoral). In the second phase, the mean ± standard deviation (SD) values found by Rajabi et al. were 10.8 ± 5.1 (intratumoral) and 16.6 ± 2.4 (peritumoral). We observed the following values: 4.80± 5.97 (intratumoral) and 15.63 ± 7.15 (peritumoral). Third-stage cases presented intratumoral values of 2.1 ± 2.3, compared to 3.682.1 ± 7.49 in our study, and peritumoral values of 8.2 ± 4.6, compared to 12.64 ± 4.63 in our study. Similar values were observed in the two studies, especially in the peritumoral areas. Atiakshin et al. [25] noticed an increased number of mast cells, mainly in the peritumoral area and in stage 2 cases. Similar results were found in our study. They found that the predominant cellular type was degranulated MCs [26], which was also observed in our study. In melanoma, a different MC phenotype with a reduced number of chymase-positive cells indicates that MCs undergo some phenotypic changes during melanoma progression, and that it is necessary to evaluate both immunohistochemical markers—mast cell tryptase and mast cell chymase. Atiakshin et al. proposed that mast cells and infiltrating lymphocytes have an inhibitory effect. Some data have shown a reduced capacity of mast cells in promoting the invasiveness of melanoma [27]. In our study, the highest values of mast cell density found in the intratumoral and peritumoral areas were as follows: 24 and 48.33 (T1), 14 and 27 (T2), 16.33 and 17.66 (T3), and 6.33 and 12.33 (T4). Significant correlations were found between intratumoral and peritumoral mast cell density in T3 malignant melanoma cases, as also reported by Juodžiukynienė et al. [28].

An important correlation has been observed between the intratumoral microvessel density (MVD) and clinical–pathological parameters—prognostic and survival—in melanoma [26,29,30,31,32]. Other studies have demonstrated that MVD does not affect the HR of OS and DFS (disease-free survival). However, a strong correlation has been observed with DFS rates at 12, 36, and 60 months [33]. In our study, uniform distribution patterns were observed in vessels and mast cells in cases from all three evaluated centers (Timișoara, Bucharest, and Sibiu).

It is well known that melanoma cells express the laminin receptor and adhere more tightly to the vessel wall [34], favoring tumor cell extravasation and metastases. Melanoma cells express pro-angiogenic factors such as FGF, VEGF, TGF, and PDGF. Their receptors are expressed by endothelial cells and activated by signals that stimulate melanoma proliferation, metastasis, and differentiation. Angiogenesis in cutaneous melanomas displayed a 69% risk of relapse and a 42% mortality rate, when compared to a 33% risk and 12% mortality rate in tumors with absent vascularity [35]. Antiangiogenic therapy (Bevacizumab, Ramucirumab, Aflibercept, Ontuxizumab, Endostatin, Sorafenib, Lenvatinib, Sunitinib, Pazopanib, Axitinib) may inhibit tumor angiogenesis. In our study, the mast cells, mainly those with a granular pattern, were found to be isolated and in small groups around the blood vessels. The highest MVD values in the intratumoral and peritumoral areas were as follows: for T1 malignant melanoma cases, 41.33 and 22.66; for T2, 24.33; for T3, 20 and 21.66; and for T4, 13.66 and 17.66. The significant correlation between MVD and mast cell density in intratumoral and peritumoral areas, and G for all stages of malignant melanoma, highlights the prognostic role of mast cells and MVD in this pathology [36].

It has been demonstrated that melanogenesis may influence the activation of hypoxia-inducible factor 1-alpha (HIF-1α), which causes melanoma progression and resistance to immunotherapy. No significant correlation was found between tumor pigmentation and disease-free survival in advanced melanoma. A “Yin and Yang” role has been suggested for melanin and active melanogenesis in melanoma diagnosis, development, progression, and therapy [37].

As research continues and offers new data regarding the complexities of angiogenesis mechanisms in melanoma, novel therapeutic approaches and personalized treatment strategies offer new horizons for the management of this aggressive malignancy. The proposal of some new reliable biomarkers of treatment response and the use of anti-angiogenic agents with conventional chemotherapy and immunotherapy may improve the diagnoses and prognoses of individuals with this type of malignancy.

Our study, as fundamental research, highlights the existence of the pronounced process of angiogenesis and an increase in the number of mast cells associated with the early stages of the disease, mainly T2. Despite the possible limitations of the present study caused by the number of cases and lack of long-term follow-up of patient progress in some of the cases, the presented observations contribute to the understanding of mast cell behavior, including their presence and interaction with other components of the tumor microenvironment and their influence on the angiogenesis process. In terms of developing combined therapies for melanoma, including anti-angiogenic therapy and immunotherapy [38,39], mast cells may constitute key therapeutic targets, particularly through the blockade of immune checkpoint inhibitors. The inhibition of mast cell activity may improve the efficiency of anti-PD-1/anti-PDL-1 therapy in melanomas and other tumor types.

## 5. Conclusions

Mast cells are promoters of intra- and peritumoral angiogenesis in the early stages. The involvement of both intratumoral and peritumoral mast cells was observed. Mast cells can be considered to be key therapeutic targets in the combined antiangiogenic and immunotherapy treatment of melanoma.

## Figures and Tables

**Table 1:** The distribution of melanoma cases according to the evaluated parameters.

T	G	Inflammatory Infiltrate	Emboli
T1	G1-28 cases	5 cases—score +1 1 case—score +2	absent
T2	G1-11 cases	3 cases—score +1 1 case—score +2 1 case—score +3 3 cases—score +1	absent
	G2-20 cases	1 case—score +2	absent
T3	G1-1 case	1 case—score 1	absent
	G2-17 cases	7 cases—score +1 1 case—score +2 3 cases—score +3	absent
	G3-3 cases	2 cases—score +1 1 case—score +2	1 case
T4	G2-11 cases	1 case—score 0 5 cases—score +1 1 case—score +2 1 case—score +3	data
	G3-1 case	absent	absent

**Table 2:** The clinical–pathological variables of melanoma cases (Bucharest Center).

No.	Breslow Depth/Clark’s Level	Ulceration Status	Histo Subtype; Growth Type	Localization	Age	Sex	LV Invasion	Tumor Stage
1	5.7 mm/V	Yes	E.S.; VGP+	Posterior thorax, excisional biopsy	75	F	No	pT4b
2	2.9 mm/IV	No	E.S.; VGP +	Posterior thorax, median line	42	F	Yes	pT3a
3	10.9 mm/IV	No	E.S.; VGP+	Posterior thorax	45	F	No	pT4a
4	1.2 mm/III	No	E.S.; VGP+	Left internal supramalleolar	35	F	No	pT2a
5	5.2 mm/III	Yes	N; exo-endophytic growth	Right shoulder/scapular area	69	F	No	pT4b
6	1.2 mm/III	No	E.S.; VGP+	Left internal supramalleolar-lateral region	35	F	No	pT2a
7	/in situ	No	In situ, lentigo malign	Left cheek	55	F	No	pT1a
8	3 mm/IV	Yes	E.S.; VGP+	Posterior thorax	40	F	No	T3
9	1.2 mm/III	No	E.S.; VGP+	Right internal supramalleolar region	40	F	No	T2
10	1.3 mm/III	No	E.S.: VGP+	Anterior thorax	39	F	No	T2
11	1.4 mm/III	No	E.S.; VGP+	Posterior thorax	41	M	No	T2
12	1.5 mm/III	No	E.S.; VGP+	Left internal supramalleolar-lateral region	42	F	No	T2
13	1.7 mm/III	No	E.S.; VGP+	Anterior thorax	40	M	No	T2
14	1.5 mm/III	No	E.S.; VGP+	Anterior thorax	39	M	No	T2
15	1.8 mm/III	No	E.S.; VGP+	Posterior thorax	37	F	No	T2
16	1.5 mm/III	No	E.S.; VGP+	Anterior thorax	38	M	No	T2
17	1.6 mm/III	No	E.S.; VGP+	Anterior thorax	35	M	No	T2
18	1.8 mm/III	No	E.S.; VGP+_	Anterior thorax	33	F	No	T2
19	1.3 mm/III	No	E.S.; VGP+_	Left shoulder/scapular area	43	F	No	T2
20	1.4 mm/III	No	E.S.; VGP+	Anterior thorax	40	M	No	T2
21	1.6 mm/III	No	E.S.; VGP+_	Arm: posterior area	45	M	No	T2
22	1.7 mm/III	No	E.S.; VGP+	Anterior thorax	50	F	No	T2

E.S.—extensive on the surface; N—nodular type; and VGP—vertical growth phase.

**Table 3:** Clinical–pathological variables of cases from Sibiu Center.

Nr Crt	Breslow Depth/Clark’s Level	Ulceration Status	Histo Subtype; Growth Type	Localization	Age	Sex	LV Invasion	Tumor Stage
1	1.1 mm/III	No	E.S.; VGP+	Thoracodorsal	64	F	0	T2
2	0/in situ	No	In situ lentigo malign	Left base of the chest, left flank	29	M	No	T1
3	0/in situ	No	In situ lentigo malign	Left arm	29	M	No	T1
4	0/in situ	No	In situ lentigo malign	Right scapular area	65	M	No	T1
5	0/in situ	No	In situ lentigo malign	Left scapular area	44	F	No	T1
6	0/in situ	No	In situ lentigo malign	Right thigh	31	F	No	T1
7	0/in situ	No	In situ lentigo malign	Left thigh	65	M	No	T1
8	0.37 mm/I	No	Lentigo malignant melanoma	Right cheek	65	M	No	T2
9	1.1 mm/III	No	E.S.; VGP+	Left thigh	38	M	No	T2
10	1.2 mm/III	No	E.S.; VGP+	Thoracodorsal	42	F	No	T2
11	1.3 mm/III	No	E.S.; VGP+	Right thigh	49	M	No	T2
12	1 mm/III	No	E.S.; VGP+	Thoracodorsal	43	M	No	T1
13	1 mm/III	No	E.S.; VGP+	Thoracodorsal	40	M	No	T1
14	0.40 mm/I	No	Lentigo malignant melanoma	Straight shoulder	43	M	No	T2
15	0.30 mm/I	No	Lentigo malignant melanoma	Interscapular vertebral	53	F	No	T1
16	0.8 mm/II	No	Lentigo malignant melanoma	Right flank	39	M	No	T1
17	1.1 mm/III	No	E.S.; VGP+	Right shoulder/scapular	40	F	No	T2
18	0.80 mm/II	No	Lentigo malignant melanoma	Left front hind leg	64	F	No	T1
19	0.30 mm/I	No	Lentigo malignant melanoma	Left hypochondrium	49	M	No	T1
20	0.40 mm/II	No	Lentigo malignant melanoma	Lumbar	48	F	No	T1

**Table 4:** Clinical–pathological variables of cases from third center (Timișoara).

No.	Breslow Depth/Clark’s Level	Ulceration	Histopathology	Localization	Age	Sex	LV Invasion	Tumor Stage
1	0.6 mm/II	No	Lentigo malignant melanoma	Left suprascapular	38	M	0	T1a
2	0.8 mm/II	No	E.S.; VGP+	Right suprascapular	31	M	0	T1
3.	1.8 mm/III	No	N; exo-endophytic growth	Right cheek	67	F	0	T3
4	1.3 mm/III	No	E.S.; VGP+	Right scapular	81	F	0	T2
5	1.5 mm/III	No	Lentigo malignant melanoma	Right genian area	60	F	0	T2
6	1 mm/II	No	Lentigo malignant melanoma	Interscapulovertebral	47	M	0	T1
7	1.3 mm/III	No	E.S.; VGP+	Thoracodorsal	38	M	0	T2
8	1.4 mm/III	No	E.S.; VGP+	Posterior thorax	43	F	0	T2
9	1 mm/II	No	Lentigo malignant melanoma	Right inguinal region	42	F	0	T1
10	1.3 mm/III	No	E.S.; VGP+	Left arm	31	F	0	T2
11	3 mm/IV	No	E.S.; VGP+	Right arm	40	M	0	T3
12	1.6 mm/III	No	E.S.; VGP+	Right arm	41	F	0	T2
13	1 mm/II	No	E.S.; VGP+	Left inguinal region	43	M	0	T1
14	1 mm/II	No	E.S.; VGP+	Left thigh	44	F	0	T1
15	5.4 mm/V	Yes	E.S; VGP+	Nasal cavity	80	F	0	T4
16	5 mm/V	Yes	E.S.; VGP+	Right thigh	82	M	0	T4
17.	4.6 mm/V	Yes	E.S.; VGP+	Right arm	85	M	0	T4
18.	4.5 mm/V	Yes	E.S.; VGP+	Left arm	83	F	0	T4
19.	4.7 mm/V	Yes	E.S.; VGP+	Left thigh	80	F	0	T4
20.	5.8 mm/V	Yes	E.S.; VGP+	Left inguinal	87	F	0	T4
21	5.5 mm/V	Yes	E.S.; VGP+	Right inguinal	88	M	0	T4
22	5.4 mm/V	Yes	E.S.; VGP+	Right thigh	80	M	0	T4
23	5.8 mm/V	Yes	E.S.; VGP+	Left thigh	79	F	0	T4
24	3 mm/IV	No	E.S.; VGP+	Left arm	42	M	0	T3
25	1.9 mm/III	No	N; exo-endophytic growth	Left cheek	69	F	0	T3
26	1 mm/II	No	E.S.; VGP+	Right thigh	43	F	0	T1
27	1 mm/II	No	E.S.; VGP+	Left arm	40	F	0	T1
28	1 mm/II	No	E.S.; VGP+	Right arm	45	F	0	T1
29	0.30 mm/II	No	Lentigo malignant melanoma	Interscapulovertebral	45	M	0	T1
30	0.4 mm/I	No	Lentigo malignant melanoma	Left suprascapular stg	43	M	0	T1
31	0.5 mm/I	No	Lentigo malignant melanoma	Left subscapular	39	F	0	T1
32	1 mm/II	No	Lentigo malignant melanoma	Interscapulovertebral	38	M	0	T1
33	1 mm/II	No	Lentigo malignant melanoma	Left arm	40	M	00	T1
34	1 mm/II	No	E.S.; VGP+	Right arm	39	F	0	T1
35	1 mm/II	No	E.S.; VGP+	Right thigh	40	F	0	T1
36	1.3 mm/III	No	E.S.; VGP+	Posterior thorax	30	F	00	T2
37	1.4 mm/III	No	E.S.; VGP+	Anterior thorax	35	F	0	T2
38	1.2 mm/III	No	E.S; VGP+	Left thigh	40	F	00	T2
39	1.5 mm/III	No	E.S.; VGP+	Right arm	40	F	0	T2
40	1.3 mm/III	No	E.S; VGP+.	Right scapular	49	F	0	T2
41	3 mm/IV	No	E.S.; VGP+	Right arm	50	F	0	T3
42	4 mm/IV	No	E.S.; VGP+	Left arm	48	M	0	T3
43	3 mm/IV	No	E.S.; VGP+	Anterior thorax	49	M	0	T3
44	4 mm/IV	No	E.S.; VGP+	Left arm	50	M	0	T3
45	2.9 mm/IV	No	E.S.; VGP +	Posterior thorax	52	M	0	T3
46	1.9 mm/III	No	N; exo-endophytic growth	Left cheek	52	M	0	T3
47	2.5 mm/IV	No	E.S.; VGP+	Left scapular	47	F	1	T3
48	3 mm/IV	No	E.S.: VGP+	Left shoulder	48	F	1	T3
49	4 mm/V	No	E.S.; VGP+	Posterior thorax	49	F	0	T3
50	4.5 mm/V	No	E.S.; VGP+	Anterior thorax	43	F	0	T3

## Data Availability

The original contributions presented in this study are included in the article. Further inquiries can be directed to the corresponding authors.
